# Investigation of the Anti-Melanogenic and Antioxidant Characteristics of *Eucalyptus camaldulensis* Flower Essential Oil and Determination of Its Chemical Composition

**DOI:** 10.3390/ijms160510470

**Published:** 2015-05-07

**Authors:** Huey-Chun Huang, Ya-Chi Ho, Jia-Min Lim, Tzu-Yun Chang, Chen-Lung Ho, Tsong-Min Chang

**Affiliations:** 1 Department of Medical Laboratory Science and Biotechnology, China Medical University, No. 91 Hsueh-Shih Road, Taichung 40402, Taiwan; E-Mail: lchuang@mail.cmu.edu.tw; 2 Department of Applied Cosmetology & Master Program of Cosmetic Sciences, Hungkuang University, No. 1018, Section 6, Taiwan Boulevard, Shalu District, Taichung 43302, Taiwan; E-Mails: tabo2545@yahoo.com.tw (Y.-C.H.); jiajia2008@hotmail.my (J.-M.L.); tzuyun1688@gmail.com (T.-Y.C.); 3 Division of Wood Cellulose, Taiwan Forestry Research Institute, No. 53, Nanhai Road, Zhongzheng District, Taipei 10066, Taiwan

**Keywords:** *Eucalyptus camaldulensis*, melanogenesis, tyrosinase, melanin, ROS

## Abstract

The effects of essential oil from *Eucalyptus camaldulensis* flowers oil on melanogenesis and the oil’s antioxidant characteristics were investigated. Assays of mushroom and cellular tyrosinase activities and melanin content of mouse melanoma cells were performed spectrophotometrically, and the expression of melanogenesis-related proteins was determined by Western blotting. The possible signaling pathways involved in essential oil-mediated depigmentation were also investigated using specific protein kinase inhibitors. The results revealed that *E. camaldulensis* flower essential oil effectively suppresses intracellular tyrosinase activity and decreases melanin amount in B16F10 mouse melanoma cells. The essential oil also exhibits antioxidant properties and effectively decreases intracellular reactive oxygen species (ROS) levels. The volatile chemical composition of the essential oil was analyzed with gas chromatography–mass spectrometry (GC/MS). The chemical constituents in the essential oil are predominately oxygenated monoterpenes (34.9%), followed by oxygenated sesquiterpenes (31.8%), monoterpene hydrocarbons (29.0%) and sesquiterpene hydrocarbons (4.3%). Our results indicated that *E. camaldulensis* flower essential oil inhibits melanogenesis through its antioxidant properties and by down-regulating both mitogen-activated protein kinases (MAPK) and protein kinase A (PKA) signaling pathways. The present study indicates that the essential oil has the potential to be developed into a skin care product.

## 1. Introduction

Melanin is responsible for skin coloration and protects the skin from damage induced by ultraviolet (UV) light. UV-induced skin hyperpigmentation causes abnormal melanin production and accumulation [1]. In the melanin production pathway, tyrosinase is the rate-limiting enzyme; this enzyme participates in the hydroxylation of L-tyrosine to L-3,4-dihydroxyphenylalanine (L-DOPA). L-DOPA is further oxidized to its corresponding *o*-quinone [2]. Several skin hyperpigmentation disorders such as freckles, age spots, melasma, post-inflammatory melanoderma and other hyperpigmentation syndromes are the result of abnormal melanin accumulation [3]. Hence, many tyrosinase inhibitors such as kojic acid [4], arbutin [5] and azelaic acid [6] are used in skin whitening products to prevent or treat abnormal skin pigmentation [7]. However, it has been reported that whitening products with chemical skin depigmenting agents can have significant side effects, including pigmented contact dermatitis caused by kojic acid [8], genotoxicity caused by arbutin [9] and transient erythema or skin irritation caused by azelaic acid [10]. Therefore, the search for an effective and safe skin depigmenting agent is still ongoing in the field of cosmetic research and development.

In the past, antioxidants have been widely used to prevent or treat disorders related to oxidative stress in the pharmaceutical and dermatological fields. Additionally, antioxidants also have been used in the cosmetic industry to delay or prevent skin aging. It has been reported that free radicals and reactive oxygen species (ROS) are associated with several diseases such as inflammation, aging and age-related diseases [11,12]. Free radical damage to the skin caused by ROS and UV-irradiation stress plays an important role in photo-aging [13,14]. Antioxidants are reported to interfere with the oxidation process by chelating oxidation-catalytic metals or by scavenging free radicals and ROS [15,16]. Hence, many antioxidants have been used to reduce oxidative stress or damage in the human body [17,18]. However, synthetic chemical antioxidants such as *tert*-butyl hydroxyanisole (BHA) and *tert*-butyl hydroxytoluene (BHT) have been reported to be carcinogenic [19]. Therefore, many studies on plant-derived antioxidants have been reported over the past decade. It has also been found that ROS can accelerate skin pigmentation. For example, nitric oxide (NO) produced by ultraviolet-irradiated keratinocytes stimulates melanin production by increasing the expression of tyrosinase and tyrosinase-related protein 1 (TRP-1) [20,21]. The contribution of ROS to melanogenesis was studied by using antioxidants such as *N*-acetyl cysteine to abolish UVB-induced melanin production [22]. Further, melanogenesis was reported to produce hydrogen peroxide (H_2_O_2_) and ROS, which place melanocytes under high-grade oxidative stress. The ROS scavengers and inhibitors of ROS generation may down-regulate UV-induced melanogenesis [23]. Hence, inhibitors of melanogenesis, antioxidants and ROS scavengers have been increasingly used in cosmetics to prevent undesirable skin hyperpigmentation [24]. The use of essential oils as functional ingredients in cosmetics is gaining momentum because of consumers’ growing interest in ingredients from natural sources. Essential oils and their components are gaining increasing interest because of widespread consumer acceptance and the potential for multipurpose functional use [25]. Recently, it has been reported that essential oils extracted from the leaves of *Aremisia argyi* [26], *Vitex negundo* Linn. [27] and *Acorus macrospadiceus* (Yamamoto) F. N. Wei et Y. K. Li. [28] show de-pigmentation activity. The increased usage of essential oils has raised a number of concerns in relation to adverse health effects which need to be addressed [29,30]. Eucalypt trees are evergreen and belong to the *Eucalyptus* genus and *Myrtaceae* family. The *Eucalyptus* genus is native to Australia and is one of the most widely planted genera in the world. The essential oils from *Eucalyptus* species have been used for pharmaceutical and medicinal purposes [31,32], and several studies have reported that essential oils from *E. camaldulensis* leaves displayed multiple pharmacological activities, including antibacterial [33] and anti-inflammatory activities [34], antitermitic activity [35], larvicidal and mosquito repellent activities [36,37], and antioxidative and antiradical activities [38]. However, so far, there have been no reports regarding potential dermatological application of essential oils from the leaves or flowers of *Eucalyptus* species. The aim of this study is to identify the chemical compositions of the essential oils extracted from *Eucalyptus camaldulensis* flowers and to determine the oils’ anti-melanogenesis activities and antioxidative characteristics.

## 2. Results and Discussion

### 2.1. Chemical Compositions of E. camaldulensis Flower Essential Oil

Hydrodistillation of the flowers of *E. camaldulensis* generated a yellow oil with a yield of 2.68 mL/100 g, based on the flower dry mass. The constituents of the oil that were identified are presented in Table 1, where all compounds are listed in order of their elution from a DB-5 non-polar column. In gas chromatography, Kovats index is used to convert retention times into system-independent constants. The retention index of a certain chemical compound is its retention time normalized to the retention times of adjacently eluting *n*-alkanes. Fifty-four compounds were identified (Table 1), representing 100% of the oil. Oxygenated monoterpenes predominated the compounds identified (34.9%), followed by oxygenated sesquiterpenes (31.8%), monoterpene hydrocarbons (29.0%), and sesquiterpene hydrocarbons (4.3%). Among the oxygenated monoterpenes, 1,8-cineole (23.9%) was the major compound, and of the oxygenated sesquiterpenes, α-eudesmol (11.6%), γ-eudesmol (8.0%), and elemol (5.0%) were the major components. γ-Terpinene (12.9%), α-pinene (6.1%), and *p*-cymene (4.9%) were the main monoterpene hydrocarbons.

Several factors are involved in determining the constituents of essential oil, including plant cultivation and/or harvesting procedures. Additionally, different analytical techniques may also result in different GC/MS data. Eucalyptol, also known as 1,8-cineole, is the major ether component in the essential oil and the major component of *E. camaldulensis* Dehn [39] As some synthetic ethers have been reported to show antioxidant activities [40], we hypothesized that eucalyptol may account for the antioxidant activity of the essential oil. Additionally, the concentration of *p*-cymene (4.9%) in the essential oil was lower than that in the leaf oils of *E. camaldulensis* var. *brevirostris* [41]. It has been found that secondary metabolites and bioactive phytoconstituents identified by GC/MS in various plants show antimicrobial, anti-inflammatory and antioxidant activities [42,43]. The chemical constituents found in *E. camaldulensis* flower essential oil may contribute significantly to the oil’s biological activity, but the biological role of the individual chemical in the essential oil still remain to be elucidated.

**Table 1:** Chemical composition of the flower oil from *Eucalyptus* *camaldulensis*.

Consituents	K.I. ^(a)^	K.I. ^(b)^	Concentration (%)	Identification ^(c)^
α-Thujene	925	930	0.6	MS, K.I., ST
α-Pinene	937	939	6.1	MS, K.I., ST
β-Pinene	976	979	0.3	MS, K.I., ST
β-Myrcene	989	990	0.3	MS, K.I., ST
α-Phellandrene	1002	1002	0.2	MS, K.I., ST
α-Terpinene	1015	1017	0.8	MS, K.I., ST
*p*-Cymene	1020	1024	4.9	MS, K.I., ST
Limonene	1025	1029	1.2	MS, K.I., ST
1,8-Cineole	1027	1031	23.9	MS, K.I., ST
*cis*-β-Ocimene	1032	1037	0.1	MS, K.I.
γ-Terpinene	1055	1059	12.9	MS, K.I., ST
Terpinolene	1086	1088	1.3	MS, K.I., ST
Linalool	1095	1096	0.1	MS, K.I., ST
3-Methyl-3-butenyl 3-methylbutanoate	1112	1114	0.5	MS, K.I.
exo-Fenchol	1120	1121	0.1	MS, K.I.
3-Methyl-2-butenyl 2-methylbutanoate	1138	1141	0.2	MS, K.I.
δ-Terpineol	1163	1166	0.0	MS, K.I.
Borneol	1167	1169	0.0	MS, K.I., ST
Terpinen-4-ol	1175	1177	5.7	MS, K.I., ST
α-Terpineol	1187	1188	3.1	MS, K.I., ST
Nerol	1228	1229	0.2	MS, K.I., ST
Methyl geranate	1323	1324	0.3	MS, K.I.
β-Elemene	1389	1390	0.2	MS, K.I., ST
( *Z*)-Jasmone	1392	1392	0.6	MS, K.I.
α-Gurjunene	1409	1409	0.1	MS, K.I.
β-Caryophyllene	1418	1419	0.9	MS, K.I., ST
Aromadendrene	1439	1441	0.1	MS, K.I., ST
*trans*-Muurola-3,5-diene	1452	1452	0.2	MS, K.I.
α-Humulene	1454	1454	0.2	MS, K.I., ST
*allo*-Aromadendrene	1458	1460	0.1	MS, K.I.
*cis*-Cadina-1(6),4-diene	1462	1463	0.3	MS, K.I.
Viridiflorene	1496	1496	0.5	MS, K.I.
α-Muurolene	1500	1500	0.3	MS, K.I.
γ-Cadinene	1512	1513	0.2	MS, K.I.
δ-Cadinene	1522	1523	0.6	MS, K.I.
*cis*-Calamenene	1528	1529	0.4	MS, K.I.
*trans*-Cadina-1,4-diene	1533	1534	0.2	MS, K.I.
Elemol	1549	1549	5.0	MS, K.I., ST
*epi*-Globulol	1555	1556	0.1	MS, K.I.
Palustrol	1567	1568	0.2	MS, K.I.
Spathulenol	1577	1578	0.2	MS, K.I., ST
Caryophyllene oxide	1582	1583	0.2	MS, K.I., ST
Globulol	1590	1590	1.0	MS, K.I., ST
Guaiol	1600	1600	0.7	MS, K.I.
*cis*-Isolongifolanone	1612	1613	0.5	MS, K.I.
1,10-di- *epi*-Cubenol	1618	1619	0.2	MS, K.I.
*iso*-Leptospermone	1622	1622	0.2	MS, K.I.
10- *epi*-γ-Eudesmol	1622	1623	0.5	MS, K.I.
1- *epi*-Cubenol	1628	1628	0.3	MS, K.I.
γ-Eudesmol	1630	1630	8.0	MS, K.I.
τ-Cadinol	1640	1640	2.6	MS, K.I.
α-Muurolol	1645	1646	0.4	MS, K.I.
α-Eudesmol	1652	1653	11.6	MS, K.I.
(2 *z*,6*z*)-Farnesol	1698	1698	0.1	MS, K.I.
Monoterpene hydrocarbons (%)			29.0
Oxygenated monoterpenes (%)			34.9
Sesquiterpene hydrocarbons (%)			4.3
Oxygenated sesquiterpenes (%)			31.8
Oil Yield (mL/100 g)			2.68 ± 0.02

^a^ Relative retention indices experimental: *n*-alkanes (C_9_–C_24_) were used as reference points in the calculation of relative retention indices; ^b^ Kovats index on a DB-5 column with reference to *n*-alkanes [1]; ^c^ MS, NIST and Wiley library spectra and the literature; K.I., Kovats index; ST, authentic standard compounds.

### 2.2. Cell Viability

**Figure 1:**
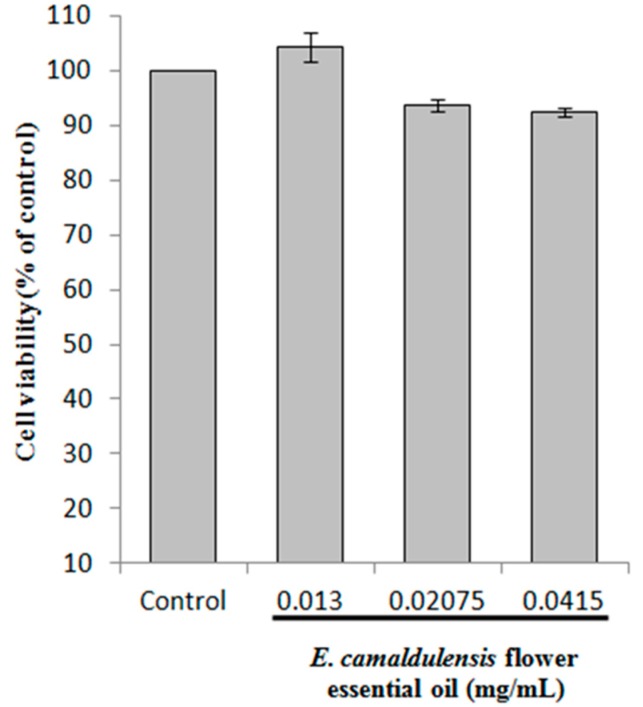
Effect of *E. camaldulensis* flower essential oil on B16F10 cell viability. Cells were treated with various concentration of essential oil (0.013, 0.02075, 0.0415 mg/mL) for 24 h, and the cell viability was measured with an MTT colorimetric assay. Results are expressed as percent cell viability relative to a control. Data are presented as the mean ± S.D.

To assess the effect of *E. camaldulensis* flower essential oil on cell viability, B16F10 mouse melanoma cells were treated with different concentrations of essential oil (0.013, 0.02075 and 0.0415 mg/mL) for 24 h. The MTT assay is a colorimetric assay for assessing cell viability. NAD(P)H-dependent cellular oxidoreductase enzymes may, under defined conditions, reflect the number of viable cells present. The results indicated that the flower essential oil had no inhibitory effect on B16F10 cell viability (Figure 1). Hence, we chose similar essential oil concentrations for our B16F10 melanoma cell experiments.

### 2.3. Inhibitory Effects of E. camaldulensis Flower Essential Oil on Melanin Production

**Figure 2:**
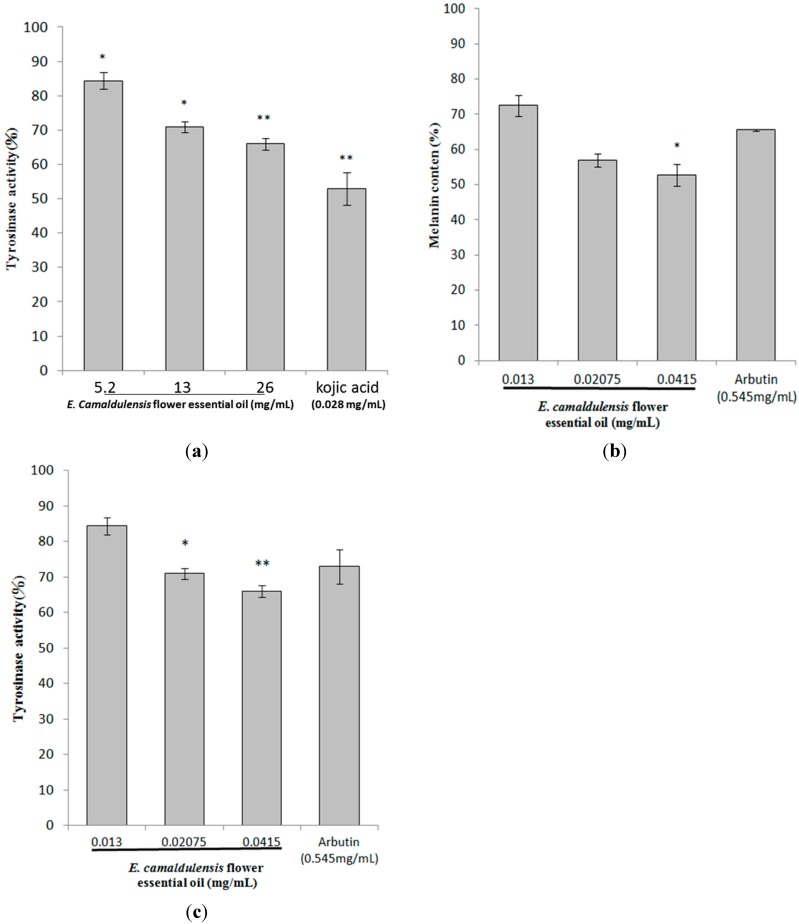
The inhibitory effects of *E. camaldulensis* flower essential oil on melanogenesis. (**a**) The effects of *E. camaldulensis* flower essential oil on mushroom tyrosinase activity; (**b**) The effects of *E. camaldulensis* flower essential oil on melanin content in B16F10 cells; (**c**) The effects of *E. camaldulensis* flower essential oil on tyrosinase activity in B16F10 cells. The results are presented as percentages of the control, and the data are presented as the mean ± S.D. of three separate experiments. Values significantly different from the control are indicated. * *p* < 0.05; ** *p* < 0.01.

Mushroom tyrosinase is widely used as the target enzyme in screening potential inhibitors of melanogenesis. The results shown in Figure 2a revealed that *E. camaldulensis* flower essential oil inhibits mushroom tyrosinase activity. Remaining enzyme activities were 82.95% ± 3.87%, 77.73% ± 5.53% and 70.04% ± 6.78% of the control for essential oil treatments of 5.2, 13 and 26 mg/mL, respectively. Tyrosinase activity was also inhibited by kojic acid, resulting in a remaining enzyme activity of 59.14% ± 1.97% of the control. Even though the concentrations of essential oil used were higher than that of kojic acid, the enzyme inhibition effect of the oil is still less than that of kojic acid. Thus, the essential oil may be a minor inhibitor of mushroom tyrosinase. The results in Figure 2b further indicated that flower essential oil significantly decreased the intracellular melanin content. The melanin content was 85.59% ± 4.39%, 74.18% ± 2.03% and 68.9% ± 1.37% for *E. camaldulensis* flower essential oil treatments of 0.013, 0.02075 and 0.0415 mg/mL, respectively. The remaining melanin content for arbutin was 77.01% ± 3.13% of the control. After treatment, the remaining intracellular tyrosinase activity was 84.38% ± 2.43%, 70.96% ± 1.55% and 65.99% ± 1.74% for *E. camaldulensis* flower essential oil treatments of 0.013, 0.02075 and 0.0415 mg/mL, respectively. The intracellular tyrosinase activity was 81.95% ± 4.78% after the cells were treated with arbutin (Figure 2c). The results shown in Figure 2c were in accordance with the results indicated in Figure 2b, which means that essential oil inhibited B16F10 intracellular tyrosinase activity and then decreased the melanin content. Those results indicated that *E. camaldulensis* flower essential oil exhibited a potent inhibitory effect on α-MSH-induced tyrosinase activity.

### 2.4. E. camaldulensis Flower Essential Oil Inhibited the Expression Levels of Melanogenesis-Related Proteins

**Figure 3:**
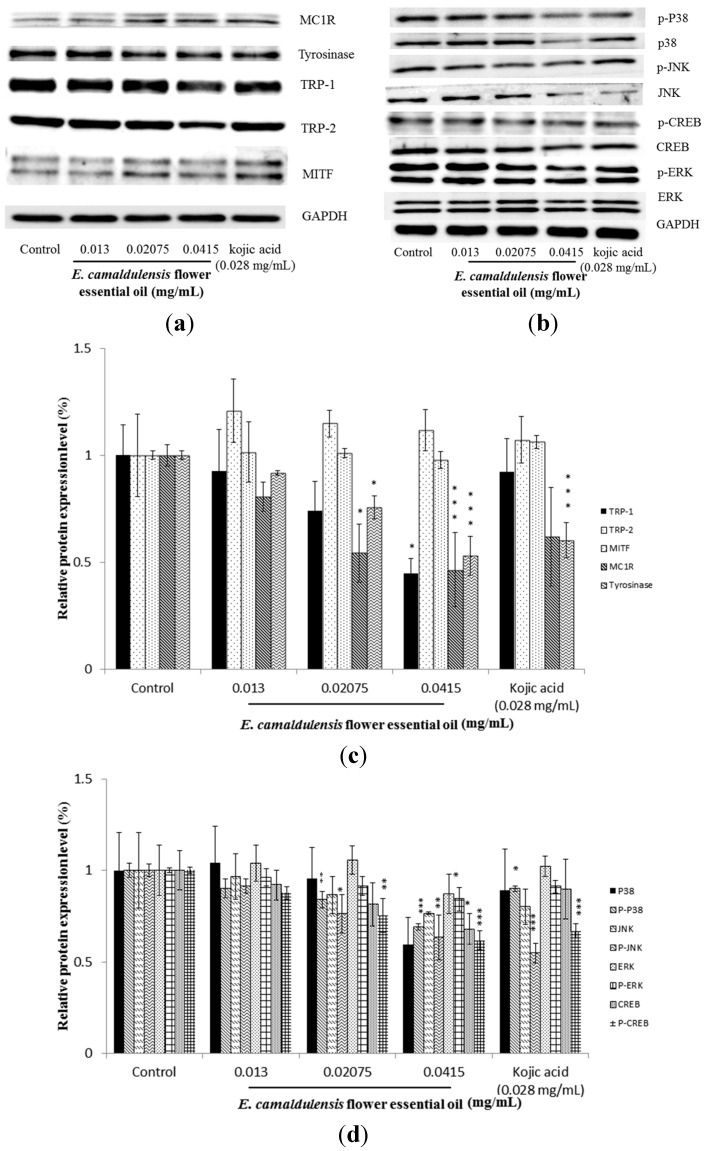
The effect of *E. camaldulensis* flower essential oil on melanogenesis-related protein expression and signaling pathways. B16F10 cells were cultured with α-MSH (100 nM) for 24 h, and then treated with various concentrations of essential oil (0.013, 0.02075, 0.0415 mg/mL) or kojic acid (200 μM) for another 24 h. The expression of cellular MITF, tyrosinase, TRP-1 and TRP-2 (**a**) or regulators of signaling pathways (**b**) was analyzed by Western blotting; The relative amounts of MITF, TRP-1, tyrosinase, TRP-2 and MC1R (**c**) or phosphorylated proteins (p-JNK, p-ERK and p-p38) (**d**) compared to the total GAPDH were calculated and analyzed using Multi Gauge 3.0 software, and the values represent the mean of triplicate experiments ± standard deviations. * *p* < 0.05; ** *p* < 0.01; *** *p* < 0.001.

The expression levels of intracellular melanogenesis-related proteins were examined using Western blots. The results presented in Figure 3a indicated that *E. camaldulensis* flower essential oil treatment led to a significantly reduced level of MC1R, tyrosinase, TRP-1 and TRP-2. However, the protein content of MITF was not significantly different after treatment. Furthermore, Figure 3b revealed that the expression levels of p38, p-p38; JNK, p-JNK; CREB, p-CREB; ERK and p-ERK were also decreased after treatment with essential oil at a concentration of 0.0415 mg/mL, which suggested that the MAPK, JNK, PKA and ERK signaling pathways are involved in *E. camaldulensis* flower essential oil-mediated inhibition of melanogenesis. In the present study, α-MSH was used as a cAMP inducer to stimulate melanin production. It is reported that α-MSH binds MC1R and activates adenylate cyclase, which catalyzes the conversion of ATP to cAMP and increases intracellular cAMP levels. This rise in intracellular cAMP levels activates cAMP-mediated signaling pathways and promotes pigmentation in skin melanocytes [44,45]. Tyrosinase, TRP-1 and TRP-2 are three major enzymes responsible for melanin biosynthesis in mammalian melanocytes [46]. MITF is the major transcriptional regulator of the tyrosinase, TRP-1 and TRP-2 genes and is known to be the most important regulator of melanocyte pigmentation [47]. The results shown in Figure 3a indicated that *E. camaldulensis* flower essential oil decreased the expression levels of MC1R, tyrosinase, TRP-1 and TRP-2, inhibiting tyrosinase activity and decreasing the melanin content in B16F10 cells. However, the expression of MITF was unchanged after treatment with essential oil. Evaluation of the gene expression level of MITF will be carried out in the near future. The decreased MC1R expression that was observed suggests that the essential oil inhibited melanogenesis induced via α-MSH-mediated intracellular cAMP up-regulation. Moreover, the results shown in Figure 3b further confirm that flower essential oil inhibited cAMP-mediated PKA signaling. It has been reported that protein kinase A (PKA) signaling is involved in melanin production. The elevation of cellular cAMP levels could activate PKA, leading to activation of CREB and MITF transcriptional activity and resulting in expression of melanogenesis-related proteins [48]. Our results shown in Figure 3b revealed decreased expression of p-CREB and CREB, suggesting that the essential oil inhibits melanin synthesis by blocking the PKA pathway. It is well known that the MAPK family comprises three types of protein kinases, including extracellular responsive kinase (ERK), c-Jun *N*-terminal kinase (JNK) and p38 MAPK. Earlier studies have reported that p38 activation positively contributes to melanin production by activating the cAMP response element-binding protein (CREB), which then activates MITF expression [49,50]. The results in Figure 3b provide evidence that the essential oil can inactivate p38 and CREB, thereby inhibiting melanin production. In the Western blotting experiments, GAPDH was used as an internal loading control. Because the GAPDH gene is often stably and constitutively expressed at high levels in most tissues and cells, it is considered a housekeeping gene. For this reason, GAPDH is commonly used as a loading control for Western blot.

### 2.5. E. camaldulensis Flower Essential Oil Down-Regulated MAPK and PKA Signaling Pathways

**Figure 4:**
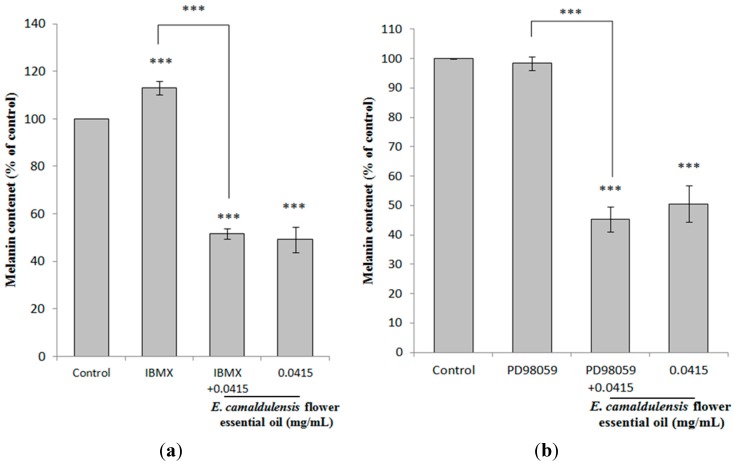

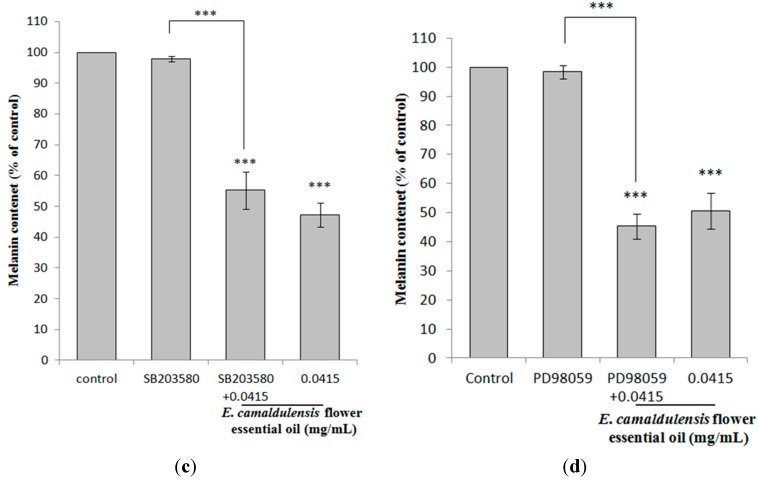
The effects of *E. camaldulensis* flower essential oil on melanin content in IBMX, PD98059-, SB203580- and SP600125-treated B16F10 cells. (**a**) The melanin content of IBMX-treated cells and (IBMX + *E. camaldulensis* flower essential oil)-treated cells; (**b**) The melanin content of PD98059-treated cells and (PD98059 + *E. camaldulensis* flower essential oil)-treated cells; (**c**) The melanin content of SB203580- and (SB203580 + *E. camaldulensis* flower essential oil)-treated cells; (**d**) The melanin content of SP600125- and (SP600125 + *E. camaldulensis* flower essential oil)-treated cells. The results are represented as percentages of the control, and the data are presented as the mean ± S.D. of three separate experiments. Values significantly different from the control are indicated. *** *p* < 0.001.

To elucidate the possible action mechanisms of the essential oil on melanin production, several protein kinase regulators of melanogenesis-related signaling pathways were tested. The application of *E. camaldulensis* flower essential oil to IBMX-treated B16F10 cells significantly decreased cellular melanin content. The remaining melanin content was 51.72% ± 2.13% of the control after treatment with IBMX and essential oil (0.0415 mg/mL). The results indicated that cAMP-mediated PKA signaling was affected by the flower essential oil (Figure 4a). The addition of *E. camaldulensis* flower essential oil in PD98059-treated B16F10 cells also decreased cellular melanin content. The remaining melanin content was 45.38% ± 4.28% of the control after treatment with PD98059 and essential oil (0.0415 mg/mL). The results shown in Figure 4b indicated that the ERK-mediated signaling pathway is involved in melanin production and was affected by *E. camaldulensis* flower essential oil treatment. To investigate the role of p38 MAPK signaling on the anti-melanogenic effect of *E. camaldulensis* flower essential oil, we employed a specific inhibitor of p38, SB203580, which blocks p38 MAPK signaling. The results in Figure 4c revealed that SB203580 attenuated α-MSH-induced melanin synthesis. The remaining melanin content was 55.19% ± 6.03% of the control after treatment with SB203580 and essential oil (0.0415 mg/mL).The addition of *E. camaldulensis* flower essential oil to SP600125-treated B16F10 cells significantly decreased the cellular melanin content. The results indicated that the JNK-mediated signaling pathway, which is involved in melanin production, was affected by *E. camaldulensis* flower essential oil. The remaining melanin content was 54.82% ± 5.79% of the control after treatment with SP600125 and the essential oil (0.0415 mg/mL) (Figure 4d).

### 2.6. Antioxidant Characteristics of E. camaldulensis Flower Essential Oil

**Figure 5:**
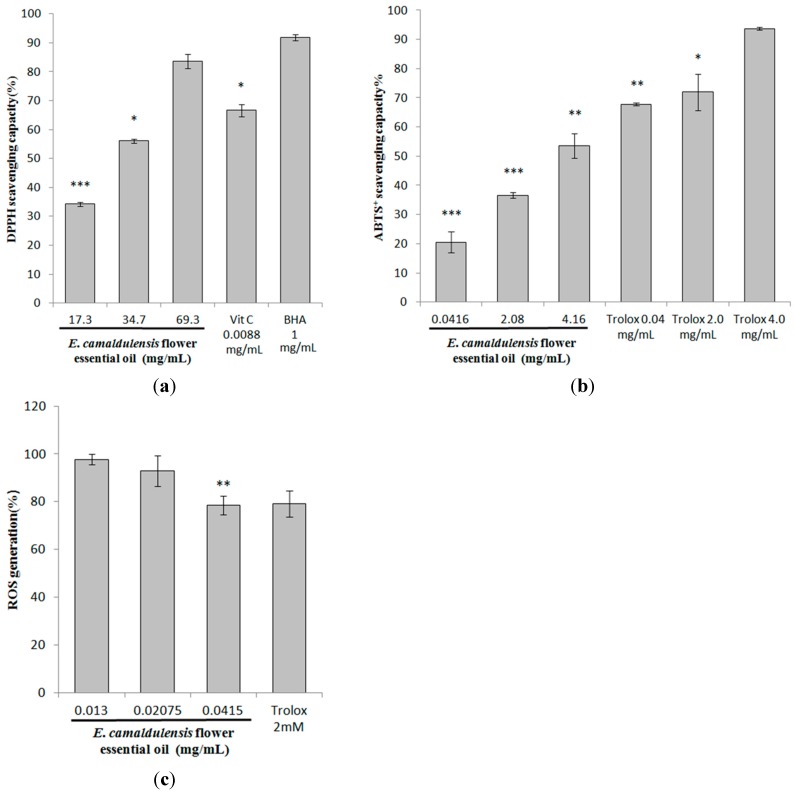
The antioxidant activities of *E. camaldulensis* flower essential oil. (**a**) DPPH scavenging activity assay; (**b**) ABTS^+^ radical scavenging capacity assay; and (**c**) determination of cellular ROS content. The results are expressed as percentages of the control. The data are presented as the mean ± S.D. * *p* < 0.05; ** *p* < 0.01; *** *p* < 0.001.

The antioxidant activity of *E. camaldulensis* flower essential oil was first measured in terms of radical scavenging ability using the DPPH assay. DPPH is a dark-colored crystalline powder composed of stable free-radical molecules. The DPPH assay is known to provide reliable information concerning the antioxidant capacity of specific compounds or extracts across a short time scale. In this study, vitamin C (0.05 mM; 0.53 mg/mL) and *tert*-butyl hydroxyanisole (BHA) (1 mg/mL) were used as positive antioxidant standards. The DPPH scavenging capacity of the oil was 34.22% ± 0.62%, 56.05% ± 0.74% and 83.58% ± 2.46% of control for essential oil concentrations of 17.3, 34.7 and 69.3 (mg/mL), respectively. In comparison, the scavenging capacities of vitamin C and BHA were 66.66% ± 2.09% and 91.89% ± 1.03%, respectively (Figure 5a).

This ABTS radical cation is blue in color and is reactive towards most antioxidants. During this reaction, the blue ABTS radical cation is converted back to its colorless neutral form. The reaction may be monitored spectrophotometrically. The ABTS^+^ assay was employed to measure the antioxidant activity of the *E. camaldulensis* flower essential oil. The ABTS^+^ scavenging capacity of the essential oil was 20.55% ± 3.54%, 36.63% ± 0.89% and 53.58% ± 4.14% of the control at concentrations of 0.0416, 2.08, 4.16 mg/mL, respectively. In contrast, the ABTS^+^ scavenging capacity of Trolox^®^ (0.04, 2 and 4 mg/mL) was 67.74% ± 0.49%, 71.93% ± 6.25%, and 93.72% ± 0.57%, respectively. The results in Figure 5b indicated that the essential oil scavenges a significant amount of ABTS^+^ free radicals. However, the flower essential oil exhibited lower ABTS^+^ radical scavenging capacity than Trolox^®^.

To further estimate the antioxidant capacity of *E. camaldulensis* flower essential oil in a cellular environment, intracellular ROS levels were determined. The concentration of H_2_O_2_ employed was 24 mM. After treatment, the remaining intracellular ROS induced by H_2_O_2_ was 78.42% ± 3.95% for 0.0415 mg/mL of the essential oil, which is similar to the value of 79.05% ± 5.44% for Trolox^®^ (2.0 mM; 0.5 mg/mL) (Figure 5c). The results shown in Figure 5c revealed that *E. camaldulensis* flower essential oil significantly suppressed intracellular ROS production only at the higher concentration of 0.0415 mg/mL. Therefore, *E. camaldulensis* flower essential oil was able to protect melanoma cells from oxidative injury by depletion of ROS generation; in addition, the oil may downregulate UV-induced melanogenesis.

To elucidate the antioxidant characteristics of *E. camaldulensis* flower essential oil, DPPH, ABTS^+^ .radical scavenging activity and ROS-scavenging capacity of the essential oil were determined as previously described [19,51]. The essential oil showed considerable antioxidant potential in all of the above analytical studies. The results demonstrated the antioxidant potential of *E. camaldulensis* flower essential oil over different ranges with distinct efficiencies. Figure 5a,b showed that the differential free radical scavenging activities of the essential oil against DPPH and ABTS^+^ radicals may result from the different mechanisms of antioxidant-radical interactions in the two assays. Furthermore, the reaction stoichiometry between the potential antioxidant chemicals in the essential oil may be different, resulting in different radical scavenging capacities [52]. It has been reported that ultraviolet irradiation induces the formation of reactive oxygen species (ROS) in cutaneous tissue, provoking toxic changes including lipid peroxidation and enzyme inactivation [53]. The results shown in Figure 5c suggested that the *E. camaldulensis* flower essential oil-induced decrease in melanin production may be attributed to depletion of cellular ROS.

## 3. Experimental Section

### 3.1. Chemicals and Reagents

Kojic acid, arbutin, Folin-Ciocalteau’s phenol reagent, 2,2'-azino-bis (3-ethylbenzthiazoline-6-sulfonic acid (ABTS), 1,1-diphenyl-2-picrylhydrazyl (DPPH), l-ascorbic acid (vitamin C), butylated hydroxyanisole (BHA), and all other chemicals and solvents were obtained from Sigma-Aldrich (St. Louis, MO, USA).

### 3.2. Plant Materials and Extraction of Essential Oils

Flowers of *E. camaldulensis* were collected in May 2011 from Lienhuachih Research Center of the Taiwan Forestry Research Institute in central Taiwan. The samples were compared with specimen no. 59916 from the Herbarium of National Ilan University (NIU). The voucher specimen (CLH-040) was deposited in the NIU herbarium. Flowers were collected for subsequent extraction and analysis. The flowers (1 kg) were first diced, then placed in a round-bottom flask and hydrodistilled for 8 h with 3 L of distilled water. The essential oil obtained was dried with anhydrous sodium sulfate. The oil yield and all test data are the average of triplicate analyses. The essential oil was collected in a sealed glass bottle and stored in a 4 °C refrigerator. In the present study, the essential oil was diluted with dimethyl sulfoxide (DMSO), and DMSO was used as a negative control in the following experiments.

### 3.3. Gas Chromatography-Mass Spectrometry (GC/MS) Analysis of Essential Oil

A Hewlett-Packard HP 6890 gas chromatograph equipped with a DB-5 fused silica capillary column (30 m × 0.25 mm × 0.25 μm film thickness, J&W Scientific, Folsom, CA, USA) and a FID detector was used for quantitative determination of oil components. Oven temperature was programmed as follows: 50 °C for 2 min, rising to 250 °C at 5 °C/min. Injector temperature: 270 °C. Carrier gas: He with a flow rate of 1 mL/min. Detector temperature: 250 °C, split ratio: 1:10. Diluted samples (1.0 μL, 1/100, *v*/*v*, in ethyl acetate) were injected manually in split mode. Identification of the oil components was based on their retention indices and mass spectra, obtained from GC/MS analysis on a Hewlett-Packard HP 6890/HP5973 equipped with a DB-5 fused silica capillary column (30 m × 0.25 mm × 0.25 μm film thickness, J&W Scientific). The GC analysis parameters listed above were used, and MS spectra were obtained (full scan mode: scan time: 0.3 s, mass range was *m*/*z* 30–500) in the EI mode at 70 eV. Data are expressed as the means ± SD of three independent experiments. Identification of the leaf essential oil constituents was based on comparisons of retention index (RI) [54], retention times (RT) and mass spectra with those obtained from authentic standards and/or the NIST and Wiley library spectra as well as the literature [54,55].

### 3.4. Assay of Mushroom Tyrosinase Activity

To determine the inhibitory effects of *E. camaldulensis* flower essential oil on mushroom tyrosinase activity, enzyme inhibition experiments were carried out in triplicate as previously described with a slight modification [56]. An aqueous solution of mushroom tyrosinase (200 units) was added to a 96-well microplate, in a total volume of 200 μL containing 5 mM l-DOPA dissolved in 50 mM phosphate buffer (pH 6.8) and the essential oil (5.2, 13 and 26 mg/mL) or kojic acid (200 μM; 0.028 mg/mL). The assay mixture was incubated at 37 °C for 30 min. After incubation, the amount of dopachrome produced in the reaction mixture was measured by spectrophotometric analysis of absorbance at 490 nm.

### 3.5. Cell Culture and Cell Viability Assay

B16F10 (ATCC CRL-6475, BCRC60031) cells were obtained from the Bioresource Collection and Research Center (BCRC), Taiwan. The cells were maintained in DMEM (Hyclone, Logan, UT, USA) supplemented with 10% fetal bovine serum and 1% antibiotics at 37 °C and 5% CO_2_ in a humidified incubator. Cell viability assays were performed using 3-(4,5-dimethylthiazol-2-yl)-2,5-diphenyl tetrazolium bromide (MTT) [57]. The cells were exposed to various concentrations of *E. camaldulensis* flower essential oil (0.013, 0.02075 and 0.0415 mg/mL) for 24 h, and the MTT solution was then added to the wells. The insoluble derivative of MTT produced by an intracellular dehydrogenase was solubilized with ethanol-DMSO (1:1 mixture solution). The absorbance of the wells at 570 nm was read using a microplate reader.

### 3.6. Measurement of Intracellular Melanin Content

Intracellular melanin content was measured as previously described with some modifications [58]. The cells were treated with α-melanocyte stimulating hormone (α-MSH) (100 nM) for 24 h and further treated with either *E. camaldulensis* flower essential oil (0.013, 0.02075 and 0.0415 mg/mL) or arbutin (2.0 mM; 0.545 mg/mL) for another 24 h. After treatment, the cells were detached by incubation in trypsin/EDTA and subsequently centrifuged at 5000× *g* for 5 min. The cell pellets were solubilized in 1 N NaOH at 60 °C for 60 min. The melanin content was assayed by absorbance at 405 nm with spectrophotometric analysis.

### 3.7. Assay of Intracellular Tyrosinase Activity

Intracellular tyrosinase activity was determined as previously described [59]. The cells were treated with α-MSH (100 nM) for 24 h, then further treated with various concentrations of *E. camaldulensis* flower essential oil (0.013, 0.02075 and 0.0415 mg/mL) or arbutin (2.0 mM; 0.545 mg/mL) for another 24 h. After treatment, the cells were washed twice with phosphate-buffered saline and homogenized with 50 mM PBS (pH 7.5) buffer containing 1.0% Triton X-100 and 0.1 mM phenylmethyl-sulfonyl fluoride (PMSF). Cellular extracts (100 μL) were mixed with freshly prepared l-DOPA solution (5.0 mM in 50 mM phosphate-buffered saline, pH 6.8) and incubated at 37 °C for 30 min. The absorbance at 490 nm was measured with a microplate reader Gen 5™ (BIO-TEK Instrument, Winooski, VT, USA) to monitor the production of dopachrome.

### 3.8. Western Blotting Assay

The cells were treated with *E. camaldulensis* flower essential oil (0.013, 0.02075 and 0.0415 mg/mL) or kojic acid (200 μM) and lysed in PBS containing proteinase inhibitors at 4 °C for 20 min. Proteins (50 μg) were resolved by SDS-polyacrylamide gel electrophoresis and electrophoretically transferred to a polyvinylidene fluoride (PVDF) filter. The nylon filter was blocked for 1 h in 5% fat-free milk in PBST buffer (PBS with 0.05% Tween-20). After a brief wash, the filter was incubated overnight at 4 °C with several antibodies; these antibodies included anti-MC1R (1:1000), anti-MITF (1:1000), anti-tyrosinase (1:2000), anti-TRP1 (1:6000), anti-TRP2 (1:1000), anti-GAPDH (1:1500), anti-P38 (1:500), anti-p-P38 (1:500), anti-JNK (1:500), anti-p-JNK (1:500), anti-CREB (1:500), anti-p-CREB (1:500), anti-ERK (1:500), anti-p-ERK (1:500). Following incubation, the filter was extensively washed in PBST buffer. Subsequent incubation with goat anti-mouse antibody (1:10,000) conjugated to horseradish peroxidase was conducted at 25 °C for 2 h. The blot was visualized using an ECL reagent. The relative amounts of expressed proteins compared to total GAPDH were analyzed using Multi Gauge 3.0 software (Fuji, Tokyo).

### 3.9. Protein Kinase Regulators Assay

The cells were treated with α-MSH (100 nM) for 24 h followed by a 1 h addition of different protein kinase regulators (10 μM), including IBMX, PD98059, SB203580 and SP600125. After these treatments, *E. camaldulensis* flower oil (0.0415 mg/mL) and the above kinase regulators were added to the cells, and cells were incubated for an additional 23 h. The melanin contents were assayed as described above.

### 3.10. DPPH Scavenging Activity Assay

The antioxidant activity of *E. camaldulensis* flower essential oil was first measured by measuring the DPPH scavenging ability [60]. Essential oil at various concentrations (17.3, 34.7 and 69.3 mg/mL) was added to 2.9 mL of DPPH (60 μM) solution. When DPPH reacts with any antioxidant in the essential oil that can donate hydrogen, it is reduced, and the resulting decrease in absorbance at 517 nm can be recorded using a UV-Vis spectrophotometer (Jasco, V-630, Tokyo, Japan). In this study, vitamin C (0.0088 mg/mL; 0.05 mM) and BHA (1 mg/mL) were used as antioxidant standards.

### 3.11. ABTS^+^ Scavenging Capacity Assay

ABTS decolorization assays were carried out as previously described [61]. This assay involves the generation of ABTS^+^ chromophores by oxidizing ABTS with potassium persulfate. The ABTS radical cation was produced by reacting 7 mM stock solution of ABTS with 2.45 mM potassium persulfate and allowing the mixture to stand in the dark for at least 6 h before use. Absorbance at 734 nm was measured 10 min after mixing of different concentrations of the *E. camaldulensis* flower essential oil (0.0416, 2.08 and 4.16 mg/mL) with 1 mL of ABTS^+^ solution. The ABTS^+^ scavenging capacity of the essential oil was compared with that of Trolox^®^ (2.0 or 4.0 mg/mL).

### 3.12. Determination of Cellular ROS Level

Cells were cultured in 24-well plates and treated with *E. camaldulensis* flower essential oil (0.013, 0.02075 and 0.0415 mg/mL) for 24 h. The cells were then incubated with 24 mM H_2_O_2_ at 37 °C for 30 min. After incubation, 2',7'-dichloro-fluorescein diacetate (DCFH-DA) was added to the wells, and the cells were cultured for an additional 30 min. The fluorescence intensities of DCF were measured at an excitation wavelength of 504 nm and an emission wavelength of 524 nm using a Fluoroskan Ascent fluorescent reader (Thermo Scientific, Vantaa, Finland). Data were analyzed using Ascent software (Thermo Scientific, Vantaa, Finland) [62].

### 3.13. Statistical Analysis

Statistical analysis of the experimental data points was performed using the ANOVA test, which was used to compare the measured data using SPSS 12.0 statistical software (SPSS Inc., Chicago, IL, USA). Differences were considered as statistically significant for *p* < 0.05.

## 4. Conclusions

**Figure 6:**
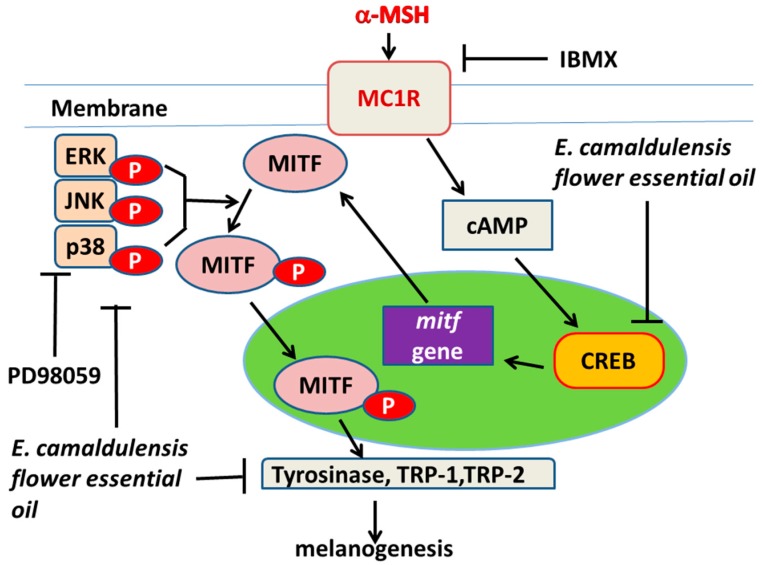
*E. camaldulensis* flower essential oil regulates melanin synthesis signaling pathways.

This is the first report regarding the effect of *E. camaldulensis* flower essential oil on melanin production. In the present study, it was determined that *E. camaldulensis* flower essential oil significantly inhibits tyrosinase activity and decreases melanin synthesis. Moreover, *E. camaldulensis* flower essential oil also exhibits intracellular free radical scavenging activity. The results suggest that *E. camaldulensis* flower essential oil decreases melanin production, likely by inhibiting the signaling pathway that regulates tyrosinase activity or by depleting cellular ROS. In addition, our results demonstrate that *E. camaldulensis* flower essential oil decreases melanogenesis in melanoma cells by inactivating PKA and MAPK signaling pathways and inhibiting tyrosinase activity (Figure 6). Additionally, the inhibitory effect of *E. camaldulensis* flower essential oil on melanin production may also be mediated by the depletion of the intracellular ROS.
